# An assessment of the experiences, and perceptions of the collateral effects of the COVID-19 lockdown measures in Southeast Nigeria: implications for policy and action

**DOI:** 10.11604/pamj.2023.46.122.36414

**Published:** 2023-12-28

**Authors:** Chigozie Jesse Uneke, Ijeoma Nkem Okedo-Alex, Bilikis Iyabo Uneke, Ifeyinwa Chizoba Akamike, Onyedika Echefu Chukwu, Irene Ifeyinwa Eze

**Affiliations:** 1 African Institute for Health Policy and Health Systems, Ebonyi State University PMB 053 Abakaliki, Abakaliki, Nigeria,; 2 Department of Community Medicine, Alex Ekwueme Federal University Teaching Hospital Abakaliki, Ebonyi State, Nigeria

**Keywords:** COVID-19, pandemic, lockdown, Nigeria

## Abstract

**Introduction:**

there is limited evidence from developing countries including Nigeria on the collateral effects of the COVID-19 lockdown on the socioeconomic lives of citizens. The aim of this study was to explore citizens´ experiences and perceptions of the impact of COVID-19 lockdown measures on daily living in Southeast Nigeria

**Methods:**

this was a cross-sectional descriptive study conducted among policymakers, researchers, non-governmental organizations (NGO) officials, and health practitioners in Southeast Nigeria. Data were collected using short message sending (SMS), emails, and key informant interviews.

**Results:**

although the COVID-19 lockdown measures had both positive and negative effects, it was largely negative. Some of the effects on family and social life were more quality time with family and improved family ties, increased social vices, reduced social and religious interaction, and disrupted academic calendars and educational pursuits. On economic life, the lockdown provided an additional source of income for those involved in the sales of facemasks and related commodities, while for others it reduced income and increased expenditures. Regarding work/career, the lockdown promoted the use of new technologies and skill acquisition, while remote work relieved work-related stress. The health effects were mostly negative including loneliness, depression, and anxiety, however, it improved health consciousness and personal hygiene. Other systemic effects stated were reduced air pollution and poor patronage at health facilities.

**Conclusion:**

without intending to, the COVID-19 lockdown in Nigeria had mixed effects on family and socioeconomic life, negatively impacting mental health but improving work-related life among others. These findings are a call to policy action to mitigate the negative effects whilst sustaining the positive gains from the lockdown.

## Introduction

The World Health Organization (WHO) categorized Nigeria as one of the 13 high-risk African countries with respect to the spread of COVID-19, largely because the country is among the vulnerable African nations, given her fragile health systems [1]. The control of COVID-19 has remained an enormous challenge for the Nigerian government and one of the broad approach to slow down the spread of the infection and prevent overwhelming the healthcare systems was by enforcing tight restrictions on population movements and a lockdown measure [2]. The lockdown in Nigeria started in March 2020 (Table 1) and was partially relaxed from early May 2020 to allow people to earn their living. This was initially restricted to high-risk areas such as Lagos and Ogun but later became total with the State governments enforcing this differentially at the State levels [3]. The lockdown involved national and interstate border closure, prohibition of public gatherings, and home confinements [3,4]. Following the full relaxation of lockdown measures by July 2020, some measures such as compulsory wearing of face masks when going outside the home, vaccination, and other WHO guidelines for preventing the further spread of COVID-19 have continued to be in place [3,5].

**Table 1:** lockdown relaxation stages in Nigeria from March to October 2020

PARAMETER	STAGE 4 March-May	STAGE 3 June-July	STAGE 2 August-September	STAGE 1 (fully relaxed) October till date
Workplaces	Essential workers only at work (20%) with mandatory use of face masks and social distancing	Approx. 75% capacity (work at home if you can)	Approx. 95% capacity use of face masks encourages	No restrictions
Places of worship	Total shut down	Total shut down	Approx. 75% capacity with social distancing	No restrictions
Banks	Essential workers only at work (20%) with mandatory use of face masks and social distancing. Reduced daily banking hours to less than 6 hours	Approx. 50% capacity with mandatory use of face mask and social distancing. Daily banking hours increased to 8 hours	Approx. 75% capacitywith mandatory use of face mask and social distancing. Daily banking hours maintained at 8 hours	100% capacity with the use of a face mask is highly encouraged
Sports facilities	Total shut down	Total shut down	Resumption of sporting activities with no fans and spectators	Sporting activities continue with no fans or spectators
Parliament	Total shut down	Approx. 75% capacity with mandatory use of face mask and social distancing.	Approx. 95% capacity use of face mask highly encouraged	100% capacity, use of face mask encouraged
Time away from home	Only for essential duties and shopping for food or health care. Dusk to dawn curfew in full force	Essential work, duties, and shopping or health care. Curfew relaxed from midnight to dawn.	No restrictions	No restrictions
Retail	Essential only open (e.g. food, doctors, logistics, drug shops, utilities)	75% of retail open, strict physical distancing, masks, etc	No restrictions, use of face masks is highly encouraged	No restrictions, and the use of face masks encouraged
Public (intra-city) transport by road	Total shut down	75% capacity with mandatory use of face mask	No restrictions, use of face masks is highly encouraged	No restrictions, and the use of face masks encouraged
Air travels	Total shut down both international and domestic	50% Resumption of local flights to only six airports with strict enforcement of the use of masks.	Resumption of all local flights to some international flight	Resumption of all local flights to much international flight
Educational institutions	Total shut down	Total shut down	Resumption of final year junior secondary and senior secondary students	All open
Gathering limits	All public gatherings suspended	All public gatherings suspended	Approx. 75% capacity, use of masks, and social distancing encouraged	No restrictions
Masks & social distancing	Mandatory on public transport, public gatherings, government offices, and any crowded environment			Mask-wearing and social distancing encouraged

In spite of the role of the lockdown measures in halting community transmission in several climes, it has been associated with a variety of collateral effects. Studies have documented various effects of the COVID-19 lockdown on social life, mental health, and daily living. Some of the positive effects stated include reduced levels of mental health issues such as anxiety, depression, and loneliness, increased communal living and social connectedness, disruptions in routines, and more time for pastimes [6]. However, some other studies have reported increased suicidal tendencies, loneliness, nervousness as well as difficulty affording food and other basic needs during the lockdowns [7-9]. Other behavioral changes include increased food and alcohol consumption, sedentariness, and sporting activities [7,10]. Some studies also reported environmental effects such as improved air quality and reduced carbon footprint [11-13]. Most of these studies provide evidence from high-income countries. There is limited evidence from developing countries including Nigeria on the effects of the lockdown on the socioeconomic lives of its citizens. The aim of this study was to explore citizens´ experiences and perceptions of the impact of COVID-19 lockdown measures on daily living in Southeast Nigeria.

## Methods

**Study area:** the study was conducted in five states (Abia, Anambra, Ebonyi, Enugu and Imo) located in South-eastern Nigeria. Nigeria is divided into six geopolitical zones with each containing a variable number of States. The Southeast geopolitical zone is largely the trade and industrial hub of the country and is largely occupied by the Ibo ethnic group.

**Study participants:** the study participants were policymakers, researchers, non-governmental organizations (NGO) officials, and health practitioners.

**Study design:** the study design is cross-sectional descriptive in nature.

**Sample size and sampling:** one hundred and twenty (120) alumni of the African Institute for Health Policy & Health Systems (AIHPHS) of Ebonyi State University who were residing in the five south-eastern Nigeria states were purposively selected to participate in the survey. They were selected based on their role in research, policymaking and practice.

**Data collection methods:** data was collected in 2020 using emails, short message sending (SMS) requests and key informant interviews. This data collection method was used because of the ease and feasibility because the survey was conducted during the lockdown. The participants were requested to state how the lockdown has affected them negatively and positively and how the lockdown can be eased towards normalcy. The SMS and emails were pretested and corrections effected before final use. The key informant interviews were held with eight interviewees namely a policymaker, parliamentarian, teacher, doctor, traditional ruler, clergy, transporter and business operator. The interview guide assessed the impact of the COVID-19 and the lockdown measures on health and socio-economic well-being and recommended strategies to exit the lockdown. Each interview was done by phone calls and took approximately 40 minutes. The phone recording function was used to record the interviews after obtaining permissions from the key informants. Prior to the interview, the key informants´ willingness to participate and their preferred time and date were ascertained via phone calls. All interviews were conducted in English language. The research researchers who were trained in qualitative research data collection carried out the interviews.

**Data analysis:** of the 120 persons contacted, 95 (79.2%) responded (91 via SMS while 4 via e-mail). Their responses were collated and presented in tables using broad themes. For the key informant interviews, the Giorgi's phenomenological approach was used to analyse the transcriptions [14,15].

**Ethical approval:** the ethical approval for this study was received from the University Research Ethics Committee of Ebonyi State University, Abakaliki, Nigeria (Reference number: EBSU/DRIC/UREC/Vol.05/072).

**Funding:** this investigation received financial support from the Alliance for Health Policy and Systems Research, WHO (AHPSR/WHO No. 2020/1038213-0; PO No. 202568286).

## Results

The respondents were made up of 8(8.8%) policymakers, 31(32.6%) medical practitioners, 45(50.5%) health researchers and 8(8.8%) NGO officials. Table 1 shows the lockdown relaxation stages in Nigeria. There were four stages of easing of the lockdown. For workplaces, at stage 4, only essential workers (20%) were permitted to go to work with mandatory use of face masks and social distancing, at stage 3, approx. 75% of workers resumed work, at stage 2, approx. 95% of workers resumed work with the use of face masks encouraged, and at stage 1, no restrictions at all. There was a total shutdown of places of worship, sports facilities, educational institutions, and public gatherings in stages 3 and 4. Essential services such as banking with mandatory use of face mask, social distancing, and reduced daily banking hours to less than 6 hours; hospitals; food, drugs, and utilities were available throughout the lockdown.

Table 2 shows the positive and negative impacts of the COVID-19 lockdown. Positive impacts on social and family life include more quality time with family and improved family ties. In economic life, there was an additional source of income for some, such as the fashion designers who had to sew facemasks. Regarding work, career, and professional development; working from home made days less stressful and increased the opportunity to learn new skills at home due to fewer outdoor meetings and activities. It also gave more time for meditation, self-evaluation, and reflection. On health and well-being, there was a reduction in the chances of contracting diseases and an improvement in consciousness of personal hygiene. Concerning environmental health, there was a reduction in air pollution from reduced industrial work and reduced use of vehicles.

**Table 2:** negative and positive impacts of the COVID-19 lockdown from the survey

Negative impact of lockdown	Positive impact of lockdown
**Social and family life**	
Reduced or even absent social & religious interaction	More quality time with family and improved family ties
The increase in the rate of poverty led to aggressiveness and an increase in social vices among the people	
Disruption of children's education	
**Economic life**	
Increase in family expenditure	Provision of additional source income to some e.g., the fashion designers who sewed face masks and people who produce sanitizers and soap.
It has reduced the movement of goods and services, thereby affecting economic activities negatively.	
Reduced my income and increased my expenses because of the price hikes	
**Work, career, self and professional development**	
The lockdown negatively affected my academic calendar.	Working from home made days less stressful
	Increased opportunity to learn new skills at home due to fewer outdoor meetings and activities
	Reduced stress and pressure of work, giving an opportunity to meditation and self-evaluation and reflection.
	Helped a lot of people to learn how to use new technologies like online meetings, training etc
	Data and information have never been as important as it is now for me.
**Health and wellbeing**	
The pandemic has affected my mental health. I am more anxious and stressed.	Reduction in the chances of contracting diseases and improvement of consciousness of personal hygiene
Lockdown deprived me of my routine exercises	
Increased anxiety over fear of infection	
Reduced social interactions lead to loneliness, depression, deviate behavior- rape, assault, violence, stealing, etc	
**Environmental Health**	
	The lockdown has helped with environmental health as in the reduction of air pollution from reduced industrial work and vehicles, also with reduced road traffic accidents
	
**Health systems**	
Exposed the weaknesses of the health systems and the impact of a lack of sufficient investment in the health sector	
Basic Health Care Services implementation activities have been slowed down due to restrictions in movement and banking services	
Reduced regular patient's hospital attendance with redundancy at my place of work	

Negative impacts on social and family life include reduced or absence of social and religious interactions, and an increase in the rate of poverty, which led to aggressiveness and social vices among the people. In economic life, there was an increase in family expenditure and a reduction in economic activities such as the import and export of goods. Regarding work and career, it affected the academic calendar negatively. Concerning health and well-being, the pandemic affected mental health with increasing anxiety, loneliness, depression, and deviant behavior such as rape, assault, violence and stealing. There were also negative impacts on the health system including exposure to the weaknesses of the health systems, basic health care services implementation activities were slowed down due to restrictions in movement and banking services, and there was a reduction in regular patient hospital attendance with redundancy at health facilities.

Table 3 summarizes the responses of key informants and shows their suggestions on how lockdown can be managed by the government. Some of these include the provision of palliatives, ensuring access to food and other social amenities such as water supply, training of healthcare workers, improving service provision and primary healthcare facilities, engaging youths to do both skilled and unskilled work, provision of soft loans, removal of taxes, among others. Table 3.1 also shows feasible strategies for exiting lockdown towards normalcy such as enforcing the wearing of facemasks, handwashing and social distancing, continuous health education, and establishment of skill acquisition centers among others. There were also suggestions on measures to monitor the abatement of COVID-19 to prevent a second wave such as continuous testing for COVID-19 for suspected cases and isolation, monitoring of public places to ensure adherence to the use of face masks, social distancing and handwashing, continuous disease surveillance, upgrading of hospitals and maintenance of isolation centers, recruitment of more human resources in the hospitals, continuous screening especially at the borders, and provision of food for the masses.

**Table 3:** summary of responses from key informant interviews

Respondent description	Demographics	Impact of COVID-19 on your health and socio-economic well-being?	Positive and negative effects of the COVID-19 lockdown on your work.	How lockdown can be managed by the Government to reduce hardship and ensure public safety.	Feasible and sustainable strategies to exit the COVID-19 lockdown towards normalcy.	Measures to monitor the abatement of COVID-19 to prevent second wave.
Policy Maker	51-60 years old, male, tertiary education, married.	Fear of being infected, cost of living increased, affected social gatherings	Positive - increased personal and environmental hygiene, - decreased risk of infection and other epidemics Negative - junior workers were not coming to work, increasing the workload, - resulted in the need to work from home, - 'I had to change office to the center of the epidemic', - reduced interaction with other healthcare workers	- by providing palliative - Provide of face mask	- maintain physical and social distancing - regular hand washing and use of face mask - avoid handshaking	-testing for COVID-19 should continue for suspected cases and isolation - monitoring of public places to ensure adherence to the use of face masks, social distancing, and hand washing
Policy Maker	51-60 years old, female, tertiary education, married.	- caused fear - caused stress because of combining work with the sensitization program - 'affected my finance'	Positive - make me more confident to go about my duties while observing the measures Negative - increase workload - it caused a decrease in funding of some projects by some donor agencies - caused restriction from carrying out fieldwork	- The government should have a system where people can access food/food reserve - adequate distribution of palliative to people - there should be proper training of healthcare workers and adequate equipment for healthcare facilities Government to improve services provided at primary health centres so that people can access services within the community without having to go long distances - increase human resources in healthcare facilities	- sustain social distancing - research into local herbs that boost immunity	-diseases surveillance should continue - A high index of suspicion should be maintained
Parliamentarian	41-50 years old, male, tertiary education, married.	- created fear - reduce regular outdoor exercise - affected finances - low income - increase the burden to take care of extended family members	Positive - improve hygiene consciousness in the workplace - reduce handshaking Negative - 'restriction of movement which prevented me from carrying oversight functions'	- establishment of facilities that can produce things like sanitizer and face mask - engaging youths in some skilled and unskilled work to reduce hardship	- hand washing and use of sanitizer should be continued - social distancing should be maintained	- facilities the government has provided should be maintained - mentorship should be encouraged to enhance sustainable development - The government should ensure that hospitals are upgraded to meet the require standards and provision of adequate consumables - increase human resources in hospitals - isolation centers should be maintained
Medical doctor	41-50 years old, male, tertiary education, married.	My mental health was affected, staying locked in for weeks on end was depressing. Extra sources of income dwindled	- The patient load has greatly reduced. -Patients are reluctant to wear masks for them to be seen	- Lockdown is not advised, Government should permit people to go about their business. -They should rather provide running water for frequent hand washing even on streets.	They should Insist and impose face masking, and establish punishment for erring citizens. -Extra provision and subsidy on transportation cost so that hike in transport coat is limited	Continuous Testing, this time at schools and churches. But most especially at the interstate borders.

**Table 3.1:** summary of responses from key informant interviews

Respondent description	Demographics	Impact of COVID-19 on your health and socio-economic well-being?	Positive and negative effects of the COVID-19 lockdown on your work.	How lockdown can be managed by the Government to reduce hardship and ensure public safety.	Feasible and sustainable strategies to exit the COVID-19 lockdown towards normalcy.	Measures to monitor the abatement of COVID-19 to prevent second wave.
**Transporter**	41-50 years old, male, secondary education, married.	The COVID-19 pandemic forced me and others to always be clean thereby preventing some diseases	Positive -It created enough time for me to relate to and monitor my family -It gave me the opportunity to review my children's academic activities. -I also used the period to embark on farm work. Negative -It shut down my business leading to poverty. -I also lost my contract and its revenue during the lockdown -The fear of contracting the virus almost made me sick as every sick person was a suspect	a. Continue creating awareness among the citizens that the virus has not been totally conquered. b. Removal of tasks on goods to enable business recovery c. Distribution of palliatives to the poorest of the poor d. Grant soft loans to business owners.	a. Some of the hygienic measures used during COVID-19 such as b. Washing of hands in public places should be sustained.	a. All immigrants should be screened at the point of entry b. Government should improve our health care centres to reduce medical tourists abroad
**Teacher**	31-40 years old, male, tertiary education, married.	Misconceptions e.g Malaria/COVID-19, wrong diagnosis, causing a lot of health harassment, fear of being wrongly diagnosed, socio-economic leading to a reduction in the value of goods and services thereby leading to an increase in the price of goods and services	i. No positive effect ii. No student update due to the COVID-19 stay at home iii. Disarrangement of the educational system e.g. semester and terminal arrangement.	a. Provision of means of Transportation e.g. interstate staff buses. b. Government should regulate the price of goods and services	Health education based on causative agents and preventive measures	Increased testing
**Businessman**	31-40 years old, male, secondary education, married.	The death rate increased to a higher level. Increase in price of commodities	There were an increase in birth rate, regulation of duties in various offices	Government should provide necessary commodities to the citizenry, churches and motor parks should be monitored by government, and palliatives should be made available to the masses.	Government should establish skill acquisition centres for the youths in various LGAs and communities. Loan and grant should be made accessible to the farmers and small scale business	Communities and village elites should rise up to educate the masses within their locality about the pandemic. Health workers should be deployed to the health centres across the country e.g doctors and nurses.
**Traditional Ruler**	41-50 years old, male, secondary education, married.	It affected me seriously based on scarcity of health officers to attend to the patients. High increase in the cost of commodities	Lots of people died and it has negative impact on educational aspect. Government should do research to avoid future occurrence.	Government should share money and food right from the grass root to upper level. Every household should be equipped with sanitizing materials	Government should educate our people on cleanness and produce qualified doctors and mid-wives in our rural places for instant primary health care and others	Government should employ qualified doctors in our health services. They should provide money and food for the masses.

## Discussion

This study explored the experiences and perceptions of the impact of COVID-19 lockdown measures on daily living in Southeast Nigeria. The COVID-19 lockdown in Nigeria had mixed effects on family and socioeconomic life, negatively impacting mental health but improving work-related life amongst others. During the lockdown, there was an opportunity for families to spend time together, and this helped to improve family ties. Family is an essential part of society and is the primary unit of socialization. The family serves the purpose of providing support for its members, however, busy schedules deprive a lot of families of this bonding, but the lockdown provided the quality time needed. Despite this positive impact on families, there were negative impacts as a result of reduced or absence of social & religious interactions, and also an increase in the rate of poverty leading to aggressiveness and social vices amongst the people. Social life and activities such as religious gatherings, group meetings, wedding ceremonies etc, have a way of distracting people from their worries and stressful days. Social ties can benefit health beyond target individuals by influencing the health of others through social networks [16].

During the lockdown, some businesses made extra income, such as the fashion designers who had to sew facemasks. These additional income opportunities could also contribute to reducing the financial stress that people face. On the other hand, there was an increase in family expenditure and a reduction in economic activities such as the import and export of goods. These negative impacts of the lockdown are areas that require attention so that during subsequent lockdowns, measures will be put in place to curtail such negative effects. Contrary to our findings, a study that analyzed the lockdown effect on economic activities in Nigeria revealed that most socioeconomic challenges including job loss, rise in poverty level, and fall in economic activities faced by individuals were not a result of the lockdown [17].

During the lockdown, working from home made work less stressful and gave more time for meditation, self-evaluation, and reflection, which can lead to better productivity and improved mental health. There was also an opportunity to learn new skills, which is essential in boosting the economic capacity of individuals. These positive impacts are necessary for career and professional development. Regarding health and well-being, the lockdown period reduced the chances of contracting diseases and improved the consciousness of personal hygiene. When people begin to take responsibility for their health, there´s a greater possibility of disease prevention [18]. The COVID-19 pandemic and lockdown instilled this consciousness of disease prevention in individuals, and this will go a long way to help in reducing the effect of further waves of COVID-19 and even other emerging diseases. On the other hand, there was a negative effect on mental health with increasing anxiety, loneliness, depression, and deviant behavior such as rape, assault, violence, stealing, etc. Other studies have also reported a negative effect on mental health [8]. Concerning environmental health, there was a reduction in air pollution from reduced industrial work and reduced use of vehicles. This is not surprising considering the amount of cars, aircraft, and industries that normally operate on a daily basis. The lockdown led to the shutting down of most of these activities. Other studies have also reported a reduction in air pollution as a result of the lockdown [11-13].

The effect of lockdown on the health system was also explored. This study showed that the weakness of the health system was exposed by the pandemic. Furthermore, Basic Health Care Services implementation activities were slowed down due to restrictions in movement and banking services, and there was redundancy at health facilities. The health system in low and middle-income countries (LMICs), including Nigeria, has been noted to be fragile and was further burdened by the COVID-19 pandemic [3]. This highlights the urgent need for strengthening the health systems in these LMICs. Some recommendations on how lockdown can be managed by the government as provided in this study include the provision of palliatives, ensuring access to food and other social amenities such as water supply, training of healthcare workers, improving service provision and primary healthcare facilities, engaging youths to do both skilled and unskilled work, provision of soft loans, removal of taxes, amongst others. Some of these measures have been demonstrated to be effective in reducing the effect of the lockdown [5].

Feasible strategies for exiting lockdown towards normalcy such as enforcing the wearing of facemasks, handwashing and social distancing, continuous health education, and establishment of skill acquisition centers amongst others were also suggested. There were also suggestions on measures to monitor the abatement of COVID-19 to prevent a second wave such as continuous testing for COVID-19 for suspected cases and isolation, monitoring of public places to ensure adherence to the use of face masks, social distancing and handwashing, continuous disease surveillance, upgrading of hospitals and maintenance of isolation centers, recruitment of more human resources in the hospitals, continuous screening especially at the borders, and provision of food for the masses. The possibility of subsequent waves of the pandemic must always be uppermost in the mind of decision-makers, and it is important to have a clear plan and preparation for such an eventuality. Decisions to ease lockdown in towns, cities, and countries should be based on a range of existing and emerging evidence.

Although the study involved a wide range of stakeholders and states, the study was carried out in only one geopolitical zone, and therefore findings may not be generalized to the other parts of the country. Findings may not fully represent all the impact of COVID-19 because of the nature of participants interviewed. Further studies should explore perspectives of the poor masses to get a clearer picture of the impact at the grassroots. The purposive selection of participants and reliance on self-reports could have introduced bias into the study.

## Conclusion

The COVID-19 pandemic had both positive and negative effects on different aspects of life in Nigeria including social and family life, economic life, health and wellbeing, and environmental health. There was a negative impact on the health care system. These findings are a call to policy action to mitigate the negative effects whilst sustaining the positive gains from the lockdown. There is also an urgent need to strengthen the health system of the country.

### What is known about this topic

- *The emergence of the COVID-19 pandemic posed a threat to socioeconomic activities globally;*
- *The lockdown was a non-pharmaceutical intervention instituted to curtail the spread of the COVID-19 pandemic;*
- *There is limited evidence from developing countries including Nigeria on the effects of lockdown on the socioeconomic lives.*

### What this study adds

- *This study found that the lockdown in Nigeria had mixed effects (positive and negative);*
- *Some positive effects were on family life, personal development, increased innovative technologies, health consciousness and personal hygiene;*
- *Negative effects included increased mental health issues, social vices, unemployment, disruption of academic activities and poor utilization of health services.*
